# An easy α-glycosylation methodology for the synthesis and stereochemistry of mycoplasma α-glycolipid antigens

**DOI:** 10.3762/bjoc.8.70

**Published:** 2012-04-24

**Authors:** Yoshihiro Nishida, Yuko Shingu, Yuan Mengfei, Kazuo Fukuda, Hirofumi Dohi, Sachie Matsuda, Kazuhiro Matsuda

**Affiliations:** 1 Chiba University, Graduate School of Advanced Integration Science, Matsudo 271-8510, Chiba, Japan; 2 M. Biotech. Co. Ltd., Setagaya-ku, Fukazawa 2-1-3-1103,Tokyo 158-0081, Japan

**Keywords:** cytoplasm membrane, glycolipid antigen, glycosylation, mycoplasma, stereochemistry

## Abstract

*Mycoplasma fermentans* possesses unique α-glycolipid antigens (GGPL-I and GGPL-III) at the cytoplasm membrane, which carry a phosphocholine group at the sugar primary (6-OH) position. This paper describes a practical synthetic pathway to a GGPL-I homologue (C_16:0_) and its diastereomer, in which our one-pot α-glycosylation method was effectively applied. The synthetic GGPL-I isomers were characterized with ^1^H NMR spectroscopy to determine the equilibrium among the three conformers (*gg*, *gt*, *tg*) at the acyclic glycerol moiety. The natural GGPL-I isomer was found to prefer *gt* (54%) and *gg* (39%) conformers around the lipid tail*,* while adopting all of the three conformers with equal probability around the sugar position. This property was very close to what we have observed with respect to the conformation of phosphatidylcholine (DPPC), suggesting that the *Mycoplasma* glycolipids GGPLs may constitute the cytoplasm fluid membrane together with ubiquitous phospholipids, without inducing stereochemical stress.

## Introduction

**Figure 1:**
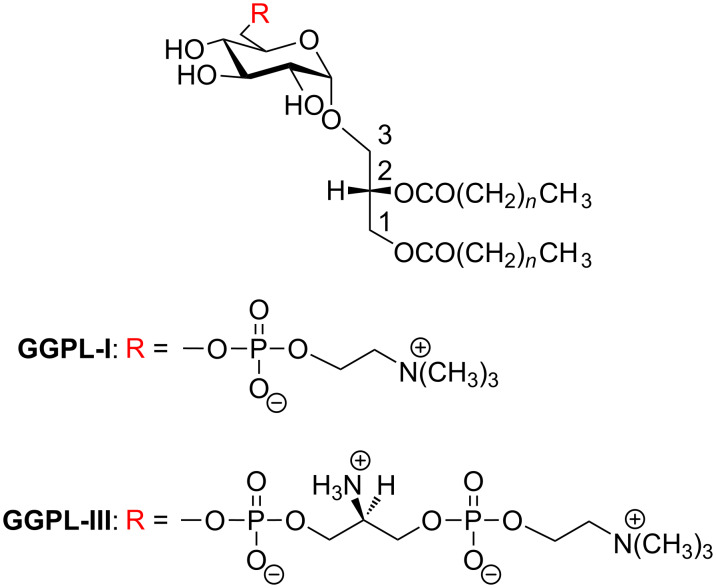
Absolute chemical structures of *M. fermentans* α-glycolipid antigens, GGPL-I and GGPl-III (GGPL: Glycosyl-*sn*-glycerophospholipid).

*Mycoplasmas* constitute a family of gram-positive microbes lacking rigid cell walls. They are suspected to be associated with human immune diseases, in either direct or indirect ways, although the molecular mechanism is not fully understood [1]. In recent biochemical studies, *Mycoplasma* outer-membrane lipoproteins [2–3] and glycolipids [4–6] are thought to serve not only as the main antigens but also as probable pathogens. Also in our research team, Matsuda et al. [7–10] isolated a new class of α-glycolipid antigens (GGPL-I and GGPL-III, Figure 1) from *M. fermentans*. Another α-glycolipid (MfGL-II), which has a chemical structure very close to GGPL-III, was identified and characterized by other groups [11–14]*.*

Absolute chemical structures of GGPL-I [15] and GGPL–III [16] have already been established by chemical syntheses of stereoisomers; these α-glycolipids have a common chemical backbone of 3-*O*-(α-D-glucopyranosyl)-*sn*-glycerol carrying phosphocholine at the sugar primary (6-OH) position. The fatty acids at the glycerol moiety are saturated, namely palmitic acid (C_16:0_) and stearic acid (C_18:0_). GGPL-I has a structural feature analogous to 1,2-di-*O*-palmitoyl phosphatidylcholine (DPPC) as a ubiquitous cell membrane phospholipid. Apparently, GGPLs are amphiphilic compounds that can form certain self-assembled structures under physiological conditions [12–13] and may give physicochemical stress on the immune system of the host [17]. In fact, our research team has proven that these α-glycolipid antigens have certain pathogenic functions [18–19].

In order to exploit their biological functions in detail, it is necessary to obtain these α-glycolipids in sufficient amounts. Thus, both genetic [20–22] and chemical synthetic approaches [23–24] are being followed, although no practical way has yet been established. In this paper, we report a chemical access to both a natural GGPL-I homologue (C_16:0_) and its diastereomer **I-a** and **I-b**, in which our one-pot α-glycosylation methodology [25–26] is effectively applied. The two GGPL-I isomers prepared thereby were characterized with ^1^H NMR spectroscopy, in terms of configuration and conformation at the asymmetric glycerol moiety.

## Results and Discussion

### A practical synthetic access to GGPL-I homologues

**Scheme 1:**
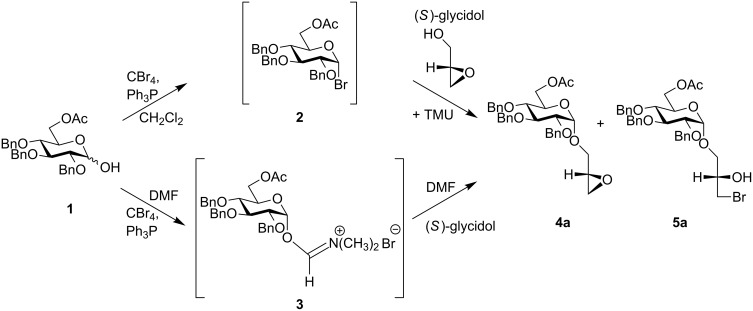
An established synthetic pathway to α-glycosyl-*sn*-glycerols **4a** and **5a**. A reagent combination of CBr_4_ and Ph_3_P (Appel–Lee reagent) is utilized in either CH_2_Cl_2_ or DMF as solvent.

GGPL-I provides two key asymmetric centers to be controlled, literally, in the synthetic pathway. One is the configuration at the chiral glycerol moiety, and another is the sugar α-glycoside linkage. In former synthetic works on 3-*O*-(α-D-glycopyranosyl)-*sn*-glycerol [27–30], chiral 1,2-*O*-isopropylidene-*sn*-glycerol has often been employed [29–30] as the acceptor substrate for different α-glycosylation reactions. In this case, however, attention should be paid to the acid-catalyzed migration of the dimethylketal group [23,29–31]. In our synthetic pathway, chiral (*S*)- or (*R*)-glycidol is employed as an alternative source of the chiral glycerol to circumvent this problem. In an established synthetic approach, 6-*O*-acetyl-2,3,4-tri-*O*-benzyl protected sugar **1** [23] is used as the donor and treated with a reagent combination of CBr_4_ and Ph_3_P (Appel–Lee reagent) in either CH_2_Cl_2_ or DMF solvent, or a mixture of the two (Scheme 1). For the reaction in CH_2_Cl_2_, *N*,*N*,*N’*,*N’*-tetramethylurea (TMU) is added after in situ formation of α-glycosyl bromide **2**, which equilibrates with a more reactive β-glycosyl bromide species [32]. In the pathway using DMF, the α-glycosylation is routed via α-glycosyl cationic imidate **3**, which was predicted in former studies [33] and evidenced in our preceding NMR and MS study [25–26].

The reaction between **1** and (*S*)-glycidol in CH_2_Cl_2_ (+ TMU) gave a mixture of epoxy compound **4a** (60–70%) and bromide **5a** (30–40%). In **5a**, the oxirane ring was opened by nucleophilic Br^−^ ions produced by Ph_3_P and CBr_4_. Also in the DMF-promoted reaction, a mixture of **4a** (70–90%) and **5a** (10–30%) was derived. In both reaction pathways, however, the glycosylation was α-selective (α:β ≥ 90:10, yields >80%) and not accompanied by isomerization at the glycerol moiety.

**Scheme 2:**
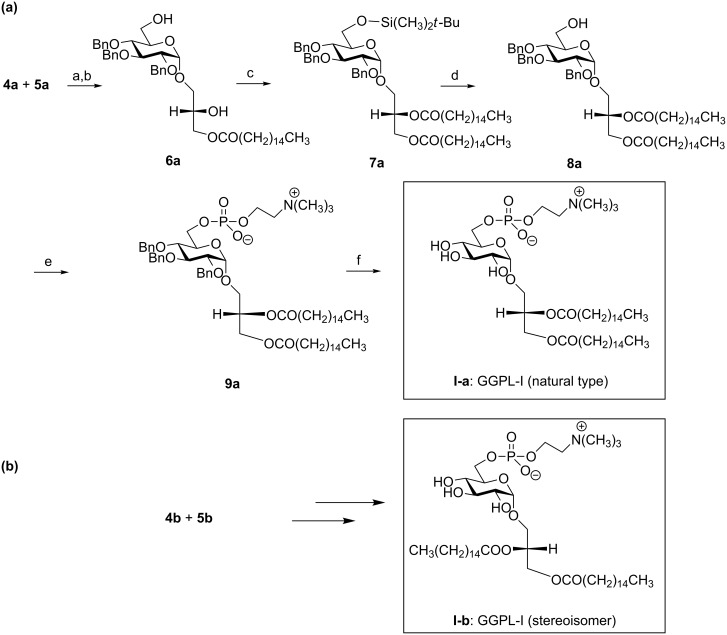
Syntheses of GGPL-I homologue **I-a** and its isomer **I-b**. Conditions: (a) K_2_CO_3_, CH_3_OH; (b) cesium palmitate in DMF; (c) TBDMS chloride then palmitoyl chloride in pyridine + DMAP; (d) TFA in CH_3_OH; (e) (i) 2-cyanoethyl-*N*,*N*,*N*’,*N*’-tetraisopropyl phosphorodiamidite, 1*H*-tetrazole and MS-4 Å in CH_2_Cl_2_; (ii) choline tosylate, 1*H*-tetrazole, (iii) *m*CPBA, (iv) aq. NH_3_ in CH_3_OH, (f) H_2_, Pd(OH)_2_/C in CH_3_OH.

A mixture of **4a** and **5a** was used in the following chemical transformation (Scheme 2). First, a *lyso*-glycolipid **6a** was derived after deprotection at the sugar hydroxymethyl position and S_N_2 substitution with cesium palmitate at the glycerol *sn-*1 position. Then, this compound was converted to glycolipid **8a** after sequential reaction of the temporary *tert*-butyldimethylsilyl (TBDMS) -protected sugar, and O-acylation at the glycerol 2-OH position to give **7a**, followed by removal of the TBDMS protecting group. For introducing the phosphocholine group at the sugar 6-OH position, we employed a phosphoroamidite method using 1*H*-tetrazole as a promoter [34]. First, **8a** was treated with 2-cyanoethyl-*N*,*N*,*N*’,*N*’-tetraisopropyl phosphorodiamidite in the presence of 1*H*-tetrazole, and then with choline tosylate to give **9a**. After removal of the sugar *O*-benzyl group by catalytic hydrogenolysis, the GGPL-I homologue **I-a** was obtained. In the same way, the GGPL-I *sn*-isomer **I-b** was derived from a mixture of **4b** and **5b** available from the reaction between **1** and (*R*)-glycidol (Scheme 1 and Scheme 2b).

### ^1^H NMR characterization of **I-a**, **I-b** and the related glycerolipids

**Figure 2:**
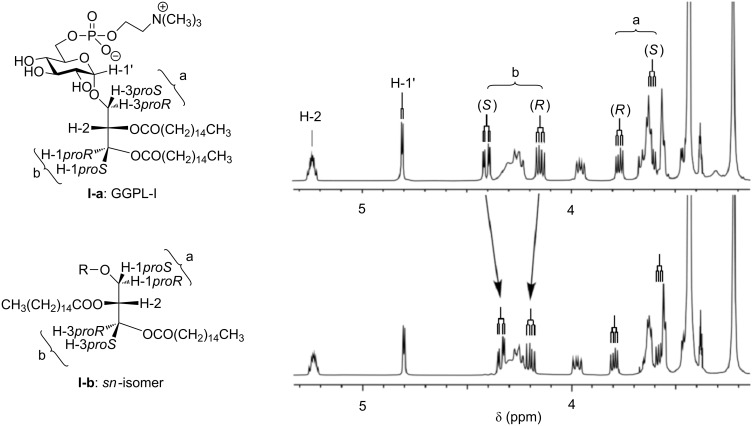
^1^H NMR spectra of **I-a** and **I-b** (500 MHz, 25 °C, CDCl_3_/CD_3_OD 10:1). The assignment of *sn*-glycerol methylene protons (H*_proR_* and H*_proS_*) was performed on the basis of our preceding studies on deuterium-labeled glycerols [35–37] and α(1→6)-linked disaccharides [38–40].

^1^H NMR spectroscopy provides a useful tool for discriminating between the two GGPL-I isomers as shown in Figure 2. A clear difference was observed in the chemical shifts of the glycerol methylene protons as designated by “a” and “b”. Conversely, little difference was observed between the *sn*-isomers at the sugar H-1 signal as well as at the glycerol H-2 (Table 1). Natural GGPL-I and GGPL-III gave ^1^H NMR data very close to those of **I-a**, indicating that both have a common skeleton of 3-*O*-(α-D-glucopyranosyl)-*sn*-glycerol [15–16].

**Table 1:** ^1^H NMR data (500 MHz) of **I-a**, **I-b**, and their precursors (**9a** and **9b**).

δ [ppm] ( ^3^*J* and ^2^*J* [Hz] )						
	
Compound	glucose	*sn*-glycerol moiety				
	
	H-1’	H-1		H-2	H-3	
	
		*proR*	*proS*		*proR*	*proS*

**I-a**^a^	4.80 (3.5)	4.14 (6.5)	4.40 (3.5, 12.0)	5.24	3.76 (5.5)	3.61 (5.5, 11.0)
**9a**	4.70 (3.5)	4.17 (6.5)	4.38 (3.0, 12.0)	5.22	3.72 (5.5)	3.52 (6.0, 11.0)
**I-b**^a^	4.80 (3.5)	3.80 (6.0)	3.57 (5.5,11.0)	5.23	4.34 (3.5)	4.20 (6.5,12.0)
**9b**	4.74 (3.5)	3.76 (5.5)	3.57 (5.5, 11.0)	5.23	4.37 (3.0)	4.19 (6.5, 12.0)

^a^These α-glycolipids were dissolved in a mixture of CDCl_3_ and CD_3_OD (10:1) at 11.2 mM concentration.

**Figure 3:**
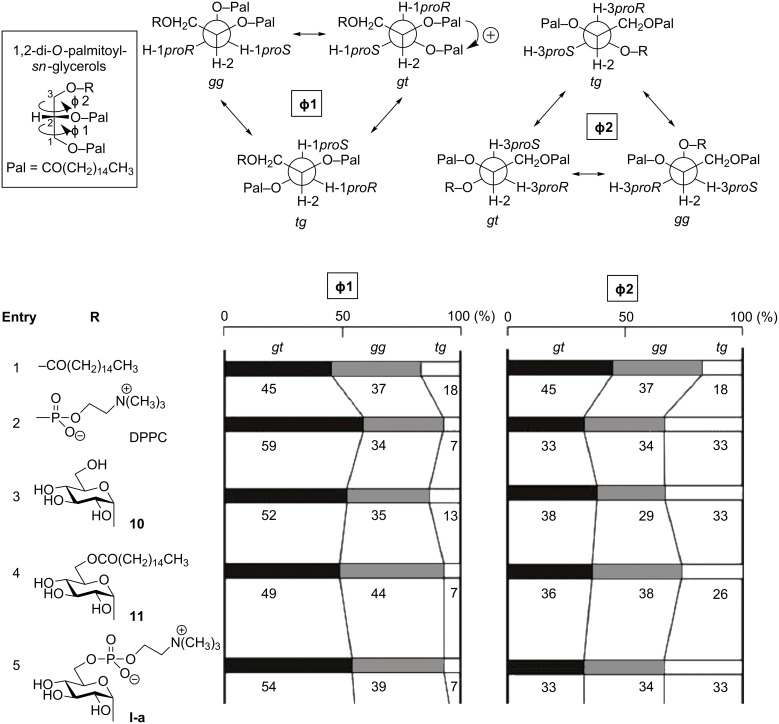
Distributions of *gg*, *gt* and *tg-*conformers in 3-substituted *sn*-glycerols at 11 mM in solutions of CDCl_3_ and CD_3_OD (10:1) at 298 K.

The glycerol moiety has two C–C single bonds. By free rotation, each of them is allowed to have three staggered conformers of *gg* (*gauche-gauche*), *gt* (*gauche-trans*) and *tg* (*trans-gauche*) (Figure 3). In solution and also in self-contacting liquid-crystalline states, these conformers are thought to equilibrate with each other. In this study, we calculated time-averaged populations of the three conformers by means of ^1^H NMR spectroscopy. As we reported in a preceding paper [41], the Karplus-type equation proposed by Haasnoot et al.[42] was adapted as follows:

2.8*gg* + 3.1*gt* + 10.7*tg* = ^3^*J*_H2,H1S_ (or ^3^*J*_H2,H3R_)

0.9*gg* + 10.7*gt* + 5.0*tg* = ^3^*J*_H2,H1R_ (or ^3^*J*_H2,H3S_) and

*gg* + *gt* + *tg* = 1.

In this equation, a perfect staggering (Φ1 and Φ2 = +60, −60 or 180 degree) is assumed for every conformer. Figure 3 summarizes the results for a series of 3-substituted 1,2-di-*O*-palmitoyl-*sn*-glycerols, which involve tripalmitin (Figure 3, entry 1), DPPC (1,2-di-*O*-palmitoylphosphatidylcholine) (Figure 3, entry 2), and GGPL-I homologues (Figure 3, entries 3–5). In a solution state with a mixture of CDCl_3_ and CD_3_OD (10:1) as the solvent and at a concentration of 11.2 mM, tripalmitin adopts the three conformers in the ratio of *gt* (45%), *gg* (37%) and *tg* (18%). In comparison with this symmetric lipid, the asymmetric phospholipid (DPPC) favors the *gt*-conformer more strongly around the tail lipid moiety along the *sn*-1,2 position, while disfavoring the *tg*-conformer, in the ratio of *gt* (59%), *gg* (34%) and *tg* (7%). The head phosphate moiety along the *sn*-2,3 position adopts the three conformers in equilibrated populations (*gg* = *gt* = *tg*).

3-*O*-(α-D-glucopyranosyl)-*sn*-glycerolipids **10** and **11** (Figure 3, entries 3 and 4) were found to have conformational properties very similar to DPPC; the lipid tail moiety prefers the *gauche* conformations (*gt* and *gg*), while the sugar moiety allows a random conformation. Here, it should be mentioned that the conformer distribution coincides between **I-a** (Figure 3, entry 5) and DPPC (Figure 3, entry 2) at the head moiety [*gt* (33%), *gg* (34%) and *tg* (33%)].

**Figure 4:**
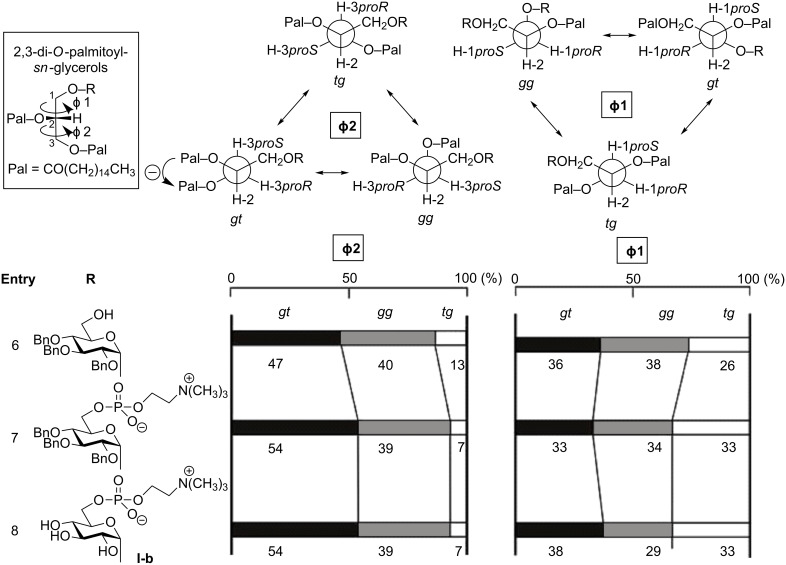
Distributions of *gg*, *gt* and *tg-*conformers in 1-substituted *sn*-glycerols. In these *sn*-isomers, Φ1 and Φ2 represent the dihedral angles around the C–C single bond at the glycerol *sn*-2,3 and 1,2-position, respectively.

**Figure 5:**
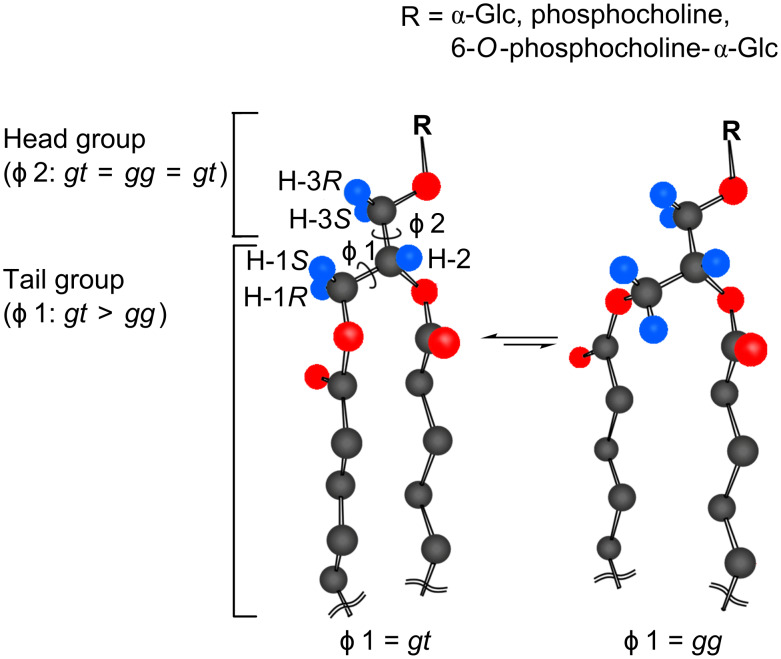
A common conformational property of GGPL-I and DPPC. The tail lipid moiety favors two *gauche*-conformers of *gt* and *gg* (*gt > gg >> tg*), while the head moiety takes three conformers in averaged populations (*gt* = *gg* = *tg*).

The above analysis was carried out also for the stereoisomer **I-b** and the related glycolipids (Figure 4, entries 6–8). The isomer (Figure 4, entry 8) showed an overall conformational property similar to **I-a** and DPPC (Figure 5), although a small difference was observed in the conformer distribution at the sugar head moiety. However, it should be recognized here that the helical direction (helicity) of the *gt* conformer in **I-b** is reversed (anticlockwise) from the case of DPPC and GGPL-I (clockwise), as depicted in Figure 3 and Figure 4.

## Conclusion

We have proposed a synthetic pathway to a GGPL-I homologue and its stereoisomer, in which our one-pot α-glycosylation methodology was effectively applied. We envisage that the simple method will allow us to prepare a variety of α-glycolipid antigens other than GGPLs and to prove their biological significance [43]. By the ^1^H NMR conformational analysis, which was based on our former studies on deuterium-labeled *sn*-glycerols and sugars, we have proven that GGPL-I and other 3-*O*-(α-D-glucopyranosyl)-*sn*-glycerolipids have a common conformational property at the chiral glycerol moiety: The lipid tail moiety prefers two *gauche*-conformations (*gg* and *gt*) in the order *gt* > *gg >> tg,* while the sugar head moiety adopts three conformers in an averaged population (*gg* = *gt* = *tg*). At the lipid tail position, the *gt-*conformer with clockwise helicity is predominant over the anticlockwise *gg*-conformer. The observed conformation was very close to what we have seen in DPPC (Figure 5). Although these results were based on the solution state in a solvent mixture of CHCl_3_ and CH_3_OH (10:1), it may be possible to assume that the mycoplasma GGPLs and the related 3-*O*-(α-D-glycopyranosyl)-*sn*-glycerolipids can constitute cytoplasm membranes in good cooperation with ubiquitous phospholipids without inducing stereochemical stress at the membrane.

The GGPL-I isomer **I-b** showed an overall conformational property similar to the natural isomer **I-a** and DPPC. However, it should be mentioned here that the chiral helicity of *gt*-conformers in **I-b** is reversed (anticlockwise) from the clockwise helicity of DPPC and GGPL-I. The difference in chirality seems critical in biological recognition events and also in physicochemical contact with other chiral constituents in cell membranes [44–45].

## Experimental

### General methods

Infrared (IR) spectra were recorded on a JASCO FT/IR-230 Fourier transform infrared spectrometer on KBr disks. All ^1^H NMR (500 MHz) spectra were recorded by using a Varian INOVA-500 or Varian Gemini 200. ^1^H chemical shifts are expressed in parts per million (δ ppm) by using an internal standard of tetramethylsilane (TMS = 0.000 ppm). Mass spectra were recorded with a JEOL JMS 700 spectrometer for fast atom bombardment (FAB) spectra. Silica gel column chromatography was performed on silica gel 60 (Merck 0.063–0.200 mm and 0.040–0.063 mm) and eluted with a mixture of toluene and ethyl acetate or a mixture of CHCl_3_ and CH_3_OH in gradient modes (100:0 to 80:20). For purification of phosphocholine-containing products, a chromatographic column packed with Iatrobeads (IATRON LABORATORIES INC., 6RS-8060) was applied and eluted with a mixture of CH_3_OH and CHCl_3_ in gradient modes. For thin-layer chromatography (TLC) analysis, Merck precoated TLC plates (silica gel 60 F_254_, layer thickness 0.25 mm) and Merck TLC aluminum roles (silica gel 60 F_254_, layer thickness 0.2 mm) were used. All other chemicals were purchased from Tokyo Kasei Kogyo Co., Ltd., Kishida Chemical Co., Ltd., and Sigma–Aldrich Chemical Company Co, Int., and were used without further purification.

**A typical procedure for the one-pot α-glycosylation:** CBr_4_ (1.6 g, 6.09 mmol) and Ph_3_P (2.02 g, 6.09 mmol) were added to a solution of 6-*O*-acetyl-2,3,4-tri-*O*-benzyl-D-glucose (**1**) (1.0 g, 2.03 mmol) in 10 mL of DMF and stirred for 3 h at rt. Then, (*S*)-glycidol (301 mg, 4.06 mmol) was added to the reaction mixture and stirred for 14 h at rt. Products were diluted with a mixture of toluene and ethyl acetate (10:1), and the solution was washed with saturated aq. NaHCO_3_ and aq. NaCl solution, dried and concentrated. The residue was purified by silica gel column chromatography in toluene and ethyl acetate to give a mixture of **4a** and **5a** (the ratio changed with reaction time) as colorless syrup. The total yield of **4a** and **5a** was between 80% and 90%.

**3-*O*-(2,3,4-tri-*O*-benzyl-α-D-glucopyranosyl)-1,2-di-*O*-palmitoyl-*sn*-glycerols 8a and 8b**: K_2_CO_3_ (379 mg, 2.74 mmol) was added to the mixture of **4a** and **5a** (1 g, 1.83 mmol based on **4a**) in CH_3_OH (20 mL) and stirred for 1 h at rt. The reaction mixture was neutralized, washed with water, dried, and concentrated. The residue was dried under reduced pressure and directly subjected to the next reaction. A mixture of caesium palmitate (2.7 g, 7.3 mmol) in DMF (40 mL) was heated at 100–110 °C, to which the DMF solution of the above residue was added slowly. The reaction mixture was stirred for 2 h at 110 °C, cooled to rt, and then filtered through a pad of Celite powder with ethyl acetate. The filtrate was washed with saturated aq. NaCl solution, dried, and concentrated. The residue was purified by silica gel column chromatography to give **6a** as a colorless syrup (830 mg, 60% yield). To a solution of **6a** (300 mg, 0.39 mmol) in pyridine (20 mL), TBDMS chloride (107 mg, 0.71 mmol) and 4-*N,N*-dimethylaminopyridine (cat.) were added. The reaction mixture was stirred for 12 h at rt, treated with methanol (2 mL) for 3 h and concentrated. The residue was purified by silica gel column chromatography in a mixture of toluene and ethyl acetate. The main product was dissolved in pyridine (20 mL) and then reacted with palmitoyl chloride (162 mg, 0.59 mmol) for 3 h at rt. The reaction mixture was treated with methanol (2 mL) and then concentrated with toluene. The residue was dissolved in a mixture of CH_3_OH and CH_2_Cl_2_ (1:1, 20 mL) and treated with trifluoroacetic acid (1 mL) for 2 h at rt. After concentration, the residue was purified by silica gel column chromatography in a mixture of toluene and ethyl acetate to give **8a** as a white waxy solid (0.32 g, 81% yield from **6a**). [α]_D_^30^ +21.1 (*c* 1.0, CHCl_3_); IR (KBr, film): 3413, 2923, 2853, 1736, 1630, 1457, 1361, 1158, 1069, 736 cm^−1^; ^1^H NMR (500 MHz, CDCl_3_): δ_H_ 7.40–7.23 (m, 5H × 3, -CH_2_C_6_*H*_5_), 5.23 (m, 1H, glycerol H-2), 4.96–4.64 (d, 2H × 3,-C*H_2_*C_6_H_5_), 4.70 (d, 1H, *J* = 3.5 Hz, H-1), 4.40 (dd, 1H, *J* = 4.0 and 12.0 Hz, glycerol H-1_proS_), 4.19 (dd, 1H, *J* = 6.0 and 12.0 Hz, glycerol H-1_proR_), 3.96 (dd, 1H, *J* = 9.5 and 9.5 Hz, H-3), 3.72 (dd, 1H, *J* = 5.5 and 10.5 Hz, glycerol H-3_proR_), 3.72 and 3.66 (b, 2H, H-6_ProR_ and H-6_proS_), 3.65 (m, 1H, H-5), 3.54 (dd, 1H, *J* = 5.5 and 10.5 Hz, glycerol H-3_proS_), 3.50 (dd, 1H, *J* = 9.5 and 10.0 Hz, H-4), 3.49 (dd, 1H, *J* = 3.5 and 9.5 Hz, H-2), 2.29 (m, 2H × 2, -OCOC*H_2_*CH_2_(CH_2_)_12_CH_3_), 1.58 (b, 2H × 2, -OCOCH_2_C*H_2_*(CH_2_)_12_CH_3_), 1.25 (b, 24H × 2, -OCOCH_2_CH_2_(C*H_2_*)_12_CH_3_), 0.88 (t, 3H × 2, *J* = 7.0 Hz, -OCOCH_2_CH_2_(CH_2_)_12_C*H_3_*); FABMS *m*/*z*: [M + Na]^+^ 1023.7.

In the same way as derived for the synthesis of **8a**, (*R*)-glycidol and **6b** (0.30 g, 0.39 mmol) was used for the synthesis of **8b** (0.30 g, 77% yield). [α]_D_^32^ +18.5 (*c* 1.0, CHCl_3_); IR (KBr, film): 3452, 2924, 2854, 1739, 1586, 1455, 1296, 1159, 1095, 710 cm^−1^; ^1^H NMR (500 MHz, CDCl_3_): δ_H_ 7.40–7.23 (m, 5H × 3, -CH_2_C_6_*H_5_*), 5.23 (b, 1H, glycerol H-2), 4.96–4.63 (d, 2H × 3,-CH_2_C_6_H_5_), 4.75 (d, 1H, *J* = 3.5 Hz, H-1), 4.38 (dd, 1H, *J* = 3.5 and 12.0 Hz, glycerol H-3_proR_), 4.21 (dd, 1H, *J* = 6.5 and 12.0 Hz, glycerol H-3_proS_), 3.96 (dd, 1H, *J* = 9.5 and 9.5 Hz, H-3), 3.73 (dd, 1H, *J* = 6.0 and 11.0 Hz, glycerol H-1_proS_), 3.76 and 3.66 (b, 2H, H-6_ProR_ and H-6_proS_), 3.65 (m, 1H, H-5), 3.57 (dd, 1H, *J* = 5.5 and 11.0 Hz, glycerol H-1_proR_), 3.51 (dd, 1H, *J* = 9.5 and 10.0 Hz, H-4), 3.50 (dd, 1H, *J* = 3.5 and 9.5 Hz, H-2), 2.29 (b, 2H × 2, -OCOC*H_2_*CH_2_(CH_2_)_12_CH_3_), 1.59 (b, 2H × 2, -OCOCH_2_C*H_2_*(CH_2_)_12_CH_3_), 1.25 (b, 24H × 2, -OCOCH_2_CH_2_(C*H_2_*)_12_CH_3_), 0.88 (t, 3H × 2, *J* = 7.0 Hz, -OCOCH_2_CH_2_(CH_2_)_12_C*H_3_*); FABMS *m*/*z*: [M + Na]^+^ 1023.7.

### A phosphorodiamidite method for the synthesis of **I-a**

(a) The reaction vessel was kept under anhydrous conditions with Ar gas in the presence of molecular sieves (50%w/w), and a solution of **8a** (0.20 g, 0.20 mmol) and 2-cyanoethyl-*N*,*N*,*N*',*N*'-tetraisopropyl phosphorodiamidite (90.4 mg, 0.30 mmol) in 10 mL of CH_2_Cl_2_ was injected. 1*H*-tetrazole (28.4 mg, 0.40 mmol) was added and stirred for 2 h at rt. Then 1*H*-tetrazole (42.6 mg, 0.60 mmol, 3.0 equiv) and choline tosylate (220.3 mg, 0.8 mmol: thoroughly dried overnight under vacuum) were added to the reaction mixture and stirred for 1.5 h at rt. The reaction was quenched by the addition of water (1 mL), and then *m*-chloroperbenzoic acid (51.8 mg, 0.3 mmol) was added at 0 °C and stirred for 10 min at rt. The reaction mixture was washed with 10 % aq. Na_2_SO_3_ solution, saturated aq. NaHCO_3_ solution, water and saturated aq. NaCl solution, dried and concentrated. The residue was dissolved in a mixture of CH_3_OH (10 mL) and 30% aq. NH_3_ (1 mL) and stirred for 15 min at rt. The reaction mixture was concentrated, and the residue was purified by column chromatography (IATROBEADS in a mixture of CHCl_3_ and CH_3_OH) to give **9a** (186 mg, 80% yield). [α]_D_^26^ +13.0 (*c* 0.45, CHCl_3_); IR (KBr, film): 3301, 2929, 2856, 2537, 1731, 1577, 1419, 1216, 1093, 925, 788, 746; ^1^H NMR (500 MHz, CDCl_3_): δ_H_ 7.35–7.25 (m, 5H × 3, -CH_2_C_6_*H_5_*), 5.22 (m, 1H, glycerol H-2), 4.93–4.61 (d, 2H × 3, -CH_2_C_6_*H_5_*), 4.70 (d, 1H, *J* = 3.5 Hz, H-1), 4.38 (dd, 1H, *J* = 3.0 and 12.0 Hz, glycerol H-1_proS_), 4.19 (b, 2H, choline -C*H_2_*CH_2_N^+^(CH_3_)_3_), 4.17 (dd, 1H, *J* = 6.5 and 12.0 Hz, glycerol H-1_proR_), 4.15 and 4.02 (m, 2H × 2, H-6_ProR_ and H-6_proS_), 3.93 (dd, 1H, *J* = 9.0 and 9.5 Hz, H-3), 3.72 (dd, 1H, *J* = 5.5 and 11.0 Hz, glycerol H-3_proR_), 3.71 (b, 1H, H-5), 3.62 (t, 1H, H-4), 3.58 (b, 2H, choline -CH_2_C*H_2_*N^+^(CH_3_)_3_), 3.52 (dd, 1H, *J* = 6.0 and 11.0 Hz, glycerol H-3_proS_), 3.46 (dd, 1H, *J* = 3.5 and 9.5 Hz, H-2), 3.15 (s, 9H, -POCH_2_CH_2_N^+^(C*H_3_*)_3_), 2.28 (m, 2H × 2, -OCOC*H_2_*CH_2_(CH_2_)_12_CH_3_), 1.58 (b, 2H × 2, -OCOCH_2_C*H_2_*(CH_2_)_12_CH_3_), 1.25 (b, 24H × 2, -OCOCH_2_CH_2_(C*H_2_*)_12_CH_3_), 0.88 (t, 3H × 2, *J* = 7.0 Hz, -OCOCH_2_CH_2_(CH_2_)_12_C*H_3_*); FABMS *m*/*z*: [M + Na]^+^ 1188.7.

(b) Compound **9a** (0.18 g, 0.15 mmol) was hydrogenated with Pd(OH)_2_/C (8 mg) under atmospheric pressure in a mixture of CH_3_OH (10 mL) and acetic acid (0.1 mL) for 7 h at rt. The reaction mixture was neutralized by the addition of Et_3_N, filtered and concentrated. The residue was purified by column chromatography with IATROBEADS (CH_3_OH and CHCl_3_) to give **I-a** (101 mg, 75% yield). [α]_D_^31^ +18.7 (*c* 1.0, CHCl_3_/CH_3_OH 10:1); IR (KBr, film): 3372, 2927, 2852, 1731, 1573, 1469, 1112, 975, 727; ^1^H NMR (500 MHz, CDCl_3_/CD_3_OD 10:1): δ_H_ 5.23 (m, 1H, glycerol H-2), 4.80 (d, 1H, *J* = 3.5 Hz, H-1), 4.39 (dd, 1H, *J* = 3.5 and 12.0 Hz, glycerol H-1_proS_), 4.30 (b, 2H, -C*H_2_*CH_2_N^+^(CH_3_)_3_), 4.24 and 3.95 (b, 2H, H-6_ProR_ and H-6_proS_), 4.14 (dd, 1H, *J* = 6.5 and 12.0 Hz, glycerol H-1_proR_), 3.75 (dd, 1H, *J* = 5.5 and 11.0 Hz, glycerol H-3_proR_), 3.67 (b, 2H, choline-CH_2_C*H_2_*N^+^(CH_3_)_3_), 3.65 (b, 1H × 2, H-3 and H-5), 3.61 (dd, 1H, *J* = 5.5 and 11.0 Hz, glycerol H-3_proS_), 3.56 (b, 1H, H-4), 3.46 (b, 1H, H-2), 3.22 (s, 9H, -POCH_2_CH_2_N^+^(C*H_3_*)_3_), 2.31 (m, 2H × 2, -OCOC*H_2_*CH_2_(CH_2_)_12_CH_3_), 1.60 (b, 2H × 2, -OCOCH_2_C*H_2_*(CH_2_)_12_CH_3_), 1.25 (b, 24H × 2, -OCOCH_2_CH_2_(C*H_2_*)_12_CH_3_), 0.88 (t, 3H × 2, *J* = 7.0 Hz, -OCOCH_2_CH_2_(CH_2_)_12_C*H_3_*); HRMS–FAB (*m*/*z*): [M + Na]^+^ calcd for C_46_H_90_NO_13_PNa, 918.6048; found, 918.6028.

In the same way as described above, **9b** (180 mg) was derived from **8b** (200 mg, 0.20 mmol) in 76% yield and converted to the GGPL-I isomer **I-b** [70 mg, 81% yield from **9b** (120 mg)]. **9b:** [α]_D_^26^ +8.1 (*c* 0.62, CHCl_3_); IR (KBr, film): 3413, 2923, 2857, 1735, 1461, 1241, 1097, 744, 495, 445; ^1^H NMR (500 MHz, CDCl_3_): δ_H_ 7.35–7.23 (m, 5H × 3, -CH_2_C_6_*H*_5_), 5.23 (b, 1H, glycerol H-2), 4.94–4.64 (d, 2H × 3,-C*H_2_*C_6_H_5_), 4.74 (d, 1H, *J* = 3.5 Hz, H-1), 4.37 (dd, 1H, *J* = 3.0 and 12.0 Hz, glycerol H-3_proR_), 4.23 (b, 2H, choline-C*H_2_*CH_2_N^+^(CH_3_)_3_), 4.19 (dd, 1H, *J* = 6.5 and 12.0 Hz, glycerol H-3_proS_), 4.16 and 4.09 (b, 2H, H-6_proR_ and H-6_proS_), 3.94 (dd, 1H, *J* = 9.5 and 9.5 Hz, H-3), 3.76 (dd, 1H, *J* = 5.5 and 11.0 Hz, glycerol H-1_proS_), 3.72 (m, 1H, H-5), 3.61 (dd, 1H, *J* = 9.0 and 9.5 Hz, H-4), 3.60 (b, 2H, choline-CH_2_C*H_2_*N^+^(CH_3_)_3_), 3.57 (dd, 1H, *J* = 5.5 and 11.0 Hz, glycerol H-1_proR_), 3.49 (dd, 1H, *J* = 3.5 and 9.5 Hz, H-2), 3.20 (s, 9H, choline-CH_2_CH_2_N^+^(C*H_3_*)_3_), 2.28 (dd, 2H × 2, *J* = 7.5 and 15 Hz, -OCOC*H_2_*CH_2_(CH_2_)_12_CH_3_), 1.58 (b, 2H × 2, -OCOCH_2_C*H_2_*(CH_2_)_12_CH_3_), 1.25 (b, 24H × 2, -OCOCH_2_CH_2_(C*H_2_*)_12_CH_3_), 0.88 (t, 3H × 2, *J* = 7.0 Hz, -OCOCH_2_CH_2_(CH_2_)_12_C*H_3_*); FABMS *m*/*z*: [M + Na]^+^ 1188.7. **I-b:** [α]_D_^31^= +10.7 (*c* 1.0, CHCl_3_/CH_3_OH 10:1); IR (KBr film): 3390, 2919, 2856, 1731, 1463, 1228, 1074, 964, 711; ^1^H NMR (500 MHz, CDCl_3_/CD_3_OD 10:1): δ_H_ 5.24 (m, 1H, glycerol H-2), 4.80 (d, 1H, *J* = 3.5 Hz, H-1), 4.34 (dd, 1H, *J* = 3.5 and 12.0 Hz, glycerol H-3_proR_), 4.28 (b, 2H, choline-C*H_2_*CH_2_N^+^(CH_3_)_3_), 4.25 and 3.97 (b, 2H, H-6_ProR_ and H-6_proS_), 4.20 (dd, 1H, *J* = 7.0 and 12.0 Hz, glycerol H-3_proS_), 3.80 (dd, 1H, *J* = 6.0 and 11.0 Hz, glycerol H-1_proS_), 3.63 (b, 1H × 2, H-3 and H-5), 3.63 (b, 2H, choline-CH_2_C*H_2_*N^+^(CH_3_)_3_), 3.58 (dd, 1H, *J* = 5.5 and 11.0 Hz, glycerol H-1_proR_), 3.55 (b, 1H, H-4), 3.46 (dd, 1H, H-2), 3.22 (s, 9H, choline-CH_2_CH_2_N^+^(C*H_3_*)_3_), 2.31 (dd, 2H × 2, *J* = 7.5 and 15 Hz, -OCOC*H_2_*CH_2_(CH_2_)_12_CH_3_), 1.60 (b, 2H × 2, -OCOCH_2_C*H_2_*(CH_2_)_12_CH_3_), 1.26 (b, 24H × 2, -OCOCH_2_CH_2_(C*H_2_*)_12_CH_3_), 0.88 (t, 3H × 2, *J* = 7.0 Hz, -OCOCH_2_CH_2_(CH_2_)_12_C*H_3_*); HRMS–FAB (*m*/*z*): M + Na]^+^ calcd for C_46_H_90_NO_13_PNa, 918.6048; found, 918.6078.

**3-*O*-(α-D-glucopyranosyl)-1,2-di-*O*-palmitoyl-*sn*-glycerol (10, entry 3)**: Compound **10** was obtained as a waxy solid (73 mg, 83% yield) from **8a** (120 mg, 0.12 mmol) by catalytic hydrogenation under the same reaction conditions as those described above for the preparation of **I-a**. [α]_D_^30^ +27.2 (*c* 1.0, CHCl_3_/CH_3_OH 10:1); IR (KBr, film): 3411, 2919, 2851, 1739, 1587, 1465, 1158, 1053, 720 cm^−1^; ^1^H NMR (500 MHz, CDCl_3_/CD_3_OD 10:1): δ_H_ 5.25 (m, 1H, glycerol H-2), 4.83 (d, 1H, *J* = 3.5 Hz, H-1), 4.40 (dd, 1H, *J* = 3.5 and 12.0 Hz, glycerol H-1_proS_), 4.16 (dd, 1H, *J* = 6.5 and 12.0 Hz, glycerol H-1_proR_), 3.82 (dd, 1H, *J* = 5.5 and 10.5 Hz, glycerol H-3_proR_), 3.78 (d, 2H, *J* = 3.5 Hz, H-6_proR_ and H-6_proS_), 3.65 (t, 1H, *J* = 9.5 and 9.5 Hz, H-3), 3.62 (dd, 1H, *J* = 6.0 and 10.5 Hz, glycerol H-3_proS_), 3.56 (dt, 1H, H-5), 3.44 (dd, 1H, *J* = 3.5 and 9.5 Hz, H-2), 3.42 (dd, 1H, *J* = 8.5 and 10.0 Hz, H-4), 2.32 (dt, 2H × 2, -OCOC*H_2_*CH_2_(CH_2_)_12_CH_3_), 1.61 (b, 2H × 2, -OCOCH_2_C*H_2_*(CH_2_)_12_CH_3_), 1.26 (b, 24H × 2, -OCOCH_2_CH_2_(C*H_2_*)_12_CH_3_), 0.88 (t, 3H × 2, *J* = 7.0 Hz, -OCOCH_2_CH_2_(CH_2_)_12_C*H_3_*); HRMS–FAB (*m*/*z*): [M + Na]^+^calcd for C_41_H_78_O_10_Na, 753.5493;found, 753.5519.

**3-*O*-(6-*O*-palmitoyl-α-D-glucopyranosyl)-1,2-di-*O*-palmitoyl-*sn*-glycerol (11, entry 4):** A mixture of **8a** (120 mg, 0.12 mmol) and palmitoyl chloride (165 mg, 0.6 mmol) in pyridine was stirred at rt for 3 h and then treated with CH_3_OH (1 mL) for 3 h. After concentration in vacuo, the residue was purified on silica gel (toluene/ethyl acetate). The main product (138 mg) was dissolved in a mixture of cyclohexene/ethanol 1:4 and subjected to catalytic hydrogenation at atmospheric pressure in the presence of Pd(OH)_2_/C (50 mg). The product was purified by silica gel column chromatography (CH_3_OH/CHCl_3_) to afford **11** (99 mg, 85% yield from **8a**). [α]_D_^30^ +20.9 (*c* 1.0, CHCl_3_/CH_3_OH 10:1); IR (KBr, film): 3414, 2919, 2851, 1739, 1605, 1465, 1375, 1176, 1054, 720 cm^−1^; ^1^H NMR (500 MHz, CDCl_3_/CD_3_OD 10:1): δ_H_ 5.25 (m, 1H, glycerol H-2), 4.82 (d, 1H, *J* = 4.0 Hz, H-1), 4.40 (dd, 1H, *J* = 3.5 and 12.0 Hz, glycerol H-1_proS_), 4.34 and 4.30 (dd × 2, 2H, *J* = 5.0 and 12.0, 2.5 and 12.0 Hz, H-6_proR_ and H-6_proS_), 4.16 (dd, 1H, *J* = 6.5 and 12.0 Hz, glycerol H-1_proR_), 3.82 (dd, 1H, *J* = 5.0 and 10.5 Hz, glycerol H-3_proR_), 3.73 (m, 1H, H-5), 3.65 (dd, 1H, *J* = 9.0 and 9.5 Hz, H-3), 3.61 (dd, 1H, *J* = 5.5 and 10.5 Hz, glycerol H-3_proS_), 3.45 (dd, 1H, *J* = 4.0 and 9.5 Hz, H-2), 3.33 (dd, 1H, *J* = 9.0 and 10.0 Hz, H-4), 2.33 (m, 2H × 3, -OCOC*H_2_*CH_2_(CH_2_)_12_CH_3_), 1.61 (b, 2H × 3, -OCOCH_2_C*H_2_*(CH_2_)_12_CH_3_), 1.26 (b, 24H × 3, -OCOCH_2_CH_2_(C*H_2_*)_12_CH_3_), 0.88 (t, 3H × 3, *J* = 7.0 Hz, -OCOCH_2_CH_2_(CH_2_)_12_C*H_3_*); HRMS–FAB (*m*/*z*): [M + Na]^+^ calcd for C_57_H_108_O_11_Na, 991.7789; found, 991.7832.
